# Nutrient Intake and Dietary Inflammatory Potential in Current and Recovered Anorexia Nervosa

**DOI:** 10.3390/nu13124400

**Published:** 2021-12-09

**Authors:** Olivia Patsalos, Bethan Dalton, Christia Kyprianou, Joseph Firth, Nitin Shivappa, James R. Hébert, Ulrike Schmidt, Hubertus Himmerich

**Affiliations:** 1 Department of Psychological Medicine, Institute of Psychiatry, Psychology & Neuroscience, King’s College London, London SE5 8AF, UK; bethan.dalton@kcl.ac.uk (B.D.); christia.kyprianou1@nhs.net (C.K.); ulrike.schmidt@kcl.ac.uk (U.S.); hubertus.himmerich@kcl.ac.uk (H.H.); 2 Division of Psychology and Mental Health, Manchester Academic Health Science Centre, University of Manchester, Manchester M13 9PL, UK; joefirth@gmail.com; 3 NICM Health Research Institute, Western Sydney University, Westmead, NSW 2145, Australia; 4 Greater Manchester Mental Health NHS Foundation Trust, Manchester Academic Health Science Centre, Manchester M25 3BL, UK; 5 Cancer Prevention and Control Program, Arnold School of Public Health, University of South Carolina, Columbia, SC 29208, USA; shivappa@email.sc.edu (N.S.); jhebert@mailbox.sc.edu (J.R.H.); 6 Department of Epidemiology and Biostatistics, Arnold School of Public Health, University of South Carolina, Columbia, SC 29208, USA; 7 Department of Nutrition, Connecting Health Innovations LLC, Columbia, SC 29201, USA; 8 South London and Maudsley NHS Foundation Trust, London SE5 8AZ, UK

**Keywords:** anorexia nervosa, dietary inflammatory index, food frequency questionnaire, inflammation, nutrient intake

## Abstract

Anorexia nervosa (AN) is characterised by disrupted and restrictive eating patterns. Recent investigations and meta-analyses have found altered concentrations of inflammatory markers in people with current AN. We aimed to assess nutrient intake in participants with current or recovered AN, as compared to healthy individuals, and explore group differences in dietary inflammatory potential as a possible explanation for the observed alterations in inflammatory markers. We recruited participants with current AN (*n* = 51), those recovered from AN (*n* = 23), and healthy controls (*n* = 49). We used the Food Frequency Questionnaire (FFQ), to calculate a Dietary Inflammatory Index (DII^®^) score and collected blood samples to measure serum concentrations of inflammatory markers. In current AN participants, we found lower intake of cholesterol, compared to HCs, and lower consumption of zinc and protein, compared to HC and recovered AN participants. A one-way ANOVA revealed no significant group differences in DII score. Multivariable regression analyses showed that DII scores were significantly associated with tumour necrosis factor (TNF)-α concentrations in our current AN sample. Our findings on nutrient intake are partially consistent with previous research. The lack of group differences in DII score, perhaps suggests that diet is not a key contributor to altered inflammatory marker concentrations in current and recovered AN. Future research would benefit from including larger samples and using multiple 24-h dietary recalls to assess dietary intake.

## 1. Introduction

Anorexia Nervosa (AN) is a severe psychiatric disorder characterised by low body weight, restrictive eating patterns, and body image disturbances. It has one of the highest standardised mortality [1,2] and relapse rates [3] of all psychiatric disorders and is frequently chronic in nature [4]. The underlying pathophysiology of AN is still poorly understood and research regarding its aetiology is ongoing. Meta-analyses have reported alterations in the immunological profile of AN patients, specifically increased concentrations of pro-inflammatory cytokines, which have been suggested as a potential contributing factor to the development and maintenance of the disorder [5,6].

Patients with AN lose weight through limiting their caloric intake and, for some, excessive physical exercise. Importantly, aside from the significantly reduced calorie intake, the macronutrient composition of their diets differs significantly from that of lean and healthy, or normal-weight people [7]. Research has shown that people with AN consume less fat, protein, and carbohydrates, but more fibre than their healthy peers [8,9]. Furthermore, it has been reported that even after treatment and weight restoration, recovered AN patients continue to exhibit suboptimal dietary intake of micronutrients and vitamins [10], as well as limited food variety [11].

Diet plays an important role in the regulation of inflammation [12] and associations between dietary patterns and inflammatory status have been reported [13]. For example, intake of dietary fibre has been associated with lower C-reactive protein (CRP), whereas consumption of saturated fatty acids has been associated with higher CRP concentrations [14,15]. It is widely accepted that the Mediterranean diet, which is generally plant-based and high in fibre and low in saturated fats, has anti-inflammatory effects and confers overall lower health risks as compared to a Western-style diet [16,17]. It also is recognised that poor nutrition significantly impacts immune function with many micronutrient deficiencies conveying profound alterations in the regulation of the immune system [18,19].

Given their highly disordered eating patterns and nutrition intake, AN patients often present with nutrient deficiencies [20]. For example, zinc deficiency has been consistently observed in AN patients and this deficiency has been associated with severe immune dysfunction, mainly affecting T-helper cells and delaying wound healing [21,22,23]. Another important nutrient is cholesterol: hypercholesterolaemia, which has been extensively studied in the context of cardiovascular disease, is frequently exhibited in people with AN and has wide ranging effects, including promoting inflammatory processes and the production of monocytes and neutrophils [24]. Crucially, sterols bind directly to several immune receptors, regulating cytokine expression [25]. It is possible, therefore, that the immunological alterations seen in AN patients could result in part from their disordered eating and patterns of nutrient intake.

In the last decade, there has been significant interest in the role of the immune system, particularly the role of cytokines in psychiatric disorders, including depression [26,27,28,29,30,31], anxiety [32,33,34], and post-traumatic disorder [35,36,37], all of which frequently co-occur with AN. Cytokines are small messenger molecules of the immune system involved in autocrine, paracrine, and endocrine signalling as well as brain functioning [38]. They are produced by a variety of cells including macrophages, as well as astrocytes and microglia [26] and have been shown to access the brain via humoral, neural, and cellular pathways [38]. In addition, they have been shown to play a role in appetite and feeding regulation via their influence on metabolic pathways and neurotransmitter signal transduction, as well as through modulating the hypothalamus-pituitary-adrenal (HPA) axis (see Himmerich et al. [29] for a review). Recent research has reported altered cytokine concentrations in AN patients compared to healthy comparison groups [39,40]. Additionally, when comparing people with current AN to those recovered from AN, significant differences in concentrations of several inflammatory markers have been reported, suggesting that some inflammatory markers could be state markers of the disorder and others trait markers of AN [41,42].

Given the evidence of cytokine alterations in AN, the reported effects of diet on inflammatory status and vice versa, and the disordered eating patterns of people with AN, we hypothesized that the documented altered inflammatory profile could result, at least partly, from their diet. Hence, primarily, we sought to compare the nutrient intakes of AN participants to people recovered from AN (recAN) and healthy controls (HC), and determine whether these groups differed in the inflammatory potential of their diet, using the Dietary Inflammatory Index (DII^®^) [43]. In addition, we explored the associations between DII scores and cytokine concentrations in participants with AN, recAN, and HC.

## 2. Materials and Methods

### 2.1. Participants

All participants were females over the age of 18 years. AN participants were required to have a current primary diagnosis of AN, according to the Diagnostic and Statistical Manual of Mental Disorders (DSM)-5 [44], and a body mass index (BMI) < 18.5 kg/m^2^. For the recAN group, participants had to (i) have previously met AN diagnostic criteria based on the DSM-IV, (ii) have maintained a BMI > 18.5 kg/m^2^ for at least 6 months prior to the study, (iii) menstruate, and (iv) have not binged, purged, or engaged in significant restrictive eating patterns for the last 3 months. Having previous alternative eating disorders diagnoses was not an exclusion criterion. AN and recAN participants were recruited via Specialist Eating Disorder Services in South London and Maudsley NHS Foundation Trust, online and poster advertisements at King’s College London (KCL), the Beat eating disorder charity’s research recruitment webpages, and through participation in other research projects. HCs were required to be of a healthy BMI (18.5–24.5 kg/m^2^) without a history of or current mental health condition, including eating disorders (EDs), and were recruited via an e-mail circular to students and staff at KCL and through online and poster advertisements. Exclusion criteria for all participants were current pregnancy and the presence of acute or chronic inflammatory conditions e.g., asthma, psoriasis, Crohn’s Disease, inflammatory bowel disease, arthritis.

Group classification (current AN, recAN or HC) was established using self-report and further assessed via a telephone screening. Screening questionnaires included the Eating Disorder Diagnostic Scale (EDDS) [45] to assess the presence of ED symptoms, and a brief inclusion/exclusion screen specific to this study, which included an assessment of physical health conditions. HC participants additionally completed the research version of the Structured Clinical Interview for DSM-IV Axis I Disorders [46] to assess the presence of current or past psychiatric disorders.

### 2.2. Measures

#### 2.2.1. Food Frequency Questionnaire

We used the European Prospective Investigation of Cancer Study (EPIC)-Norfolk Food Frequency Questionnaire (EPIC-Norfolk FFQ) [47] to collect information on the average intake (e.g., frequency, portion size) of foods and beverages during the previous year. The EPIC-Norfolk FFQ [47,48] is a semi-quantitative self-report questionnaire and requests information on 290 foods. The food list in the EPIC-Norfolk FFQ is based on items from an FFQ widely used in the US [47,49], but it was modified to reflect differences in American versus UK food items and brand names. The questionnaire consists of two parts. Part 1 is a food list of 130 lines and the lines are either individual foods, combinations of individual foods or food types. Each line also has a portion size attached to it, which is a medium serving, standard unit or household measure. Respondents select an appropriate frequency of consumption for their average intake over the last year for each line. They can select from nine frequency categories ranging from “Never or less than once a month” to “6+ per day”. Part 2 consists of several questions that ask for more detailed information about certain food lines in Part 1 (e.g., breakfast cereals). The EPIC-Norfolk FFQ has been widely used to assess dietary intake in large populations [48] and extensively validated [48,50].

We used the FFQ EPIC Tool for Analysis (FETA), an open source, cross-platform software tool, to convert EPIC-Norfolk FFQ data into nutrient and food group values [51]. Data were entered into a purposively designed comma-separated data input file following coding instructions (http://www.srl.cam.ac.uk/epic/epicffq/websitedocumentation.shtml) (accessed on 10 February 2020), which we then uploaded to FETA. The output from FETA provides an average daily nutrient and food group intake for an individual from all FFQ foods consumed; specifically, intake data for 46 nutrients and 14 basic food groups. This software produces similar nutrient and food group values to a previously validated, but less accessible tool (Compositional Analyses from Frequency Estimates (CAFÉ)) designed for converting EPIC-Norfolk FFQ data [52].

#### 2.2.2. Dietary Inflammatory Index

We used the data from the EPIC-Norfolk FFQ, as calculated by FETA, to calculate the Dietary Inflammatory Index (DII) [43]. The DII is literature-derived, using a large-scale meta-analytic strategy to compute averaged inflammatory/anti-inflammatory effects for individual nutrient parameters that have sufficient evidence to capture their effect on inflammatory markers. The DII has been validated against several peripheral markers of inflammation, including interleukin (IL)-6 [53,54] and tumour necrosis factor (TNF)-α [55].

In the current study, the following 25 food and nutrient parameters were used: alcohol, β-carotene, total carbohydrate, cholesterol, fibre, iron, folate, energy, magnesium, niacin (vitamin B_3_), total protein, retinol (vitamin A), riboflavin (vitamin B_2_), selenium, thiamine (vitamin B_1_), vitamin B_12_, vitamin B_6_, vitamin C, vitamin D, vitamin E, total fat, MUFA, PUFA, and SFA. For full details, on the steps to calculate the DII^®^ [43], see Shivappa et al. [43]. Briefly, values of the nutrients listed above are standardised into a *z* score by subtracting the mean from the global database and dividing this by the standard deviation from the global database (global daily mean intakes and standard deviations listed in Shivappa et al. [43] for each nutrient). To minimise the effect of ‘right skewing’ this value is converted into a percentile score. To achieve a symmetrical distribution with values centred on 0 (null) and bounded between −1 (maximally anti-inflammatory) and +1 (maximally pro-inflammatory), each percentile score is then doubled and ‘1’ is subtracted. The centred percentile value for each food parameter is then multiplied by its respective ‘overall food parameter-specific inflammatory effect score’, listed in Shivappa et al. [43], to obtain the ‘food parameter-specific DII score’. Finally, all of the ‘food parameter-specific DII scores’ for the available nutrients are summed to create the ‘overall DII score’ for an individual. A higher DII score indicates greater inflammatory potential of an individual’s diet. The DII score could range from +7.98 (maximally pro-inflammatory) to −8.87 (maximally anti-inflammatory) when calculated from all 45 food parameters for which the creators of the DII calculated an inflammatory score [56].

#### 2.2.3. Blood Sampling and Inflammatory Marker Quantification

To quantify inflammatory marker concentrations, blood samples were collected by trained phlebotomists. Serum was separated by centrifugation and stored at −80 °C prior to use. All samples were anonymised and stored under secure conditions. Serum was thawed at room temperature for use. The concentrations of 36 cytokines were quantified simultaneously using multiplex ELISA-based technology provided by the Meso Scale Discovery V-PLEX Plus Human Biomarker 36-Plex Kit, following the manufacturer’s instructions (Meso Scale Discovery, Rockville, MD, USA). Seven-point standard curves were run in duplicate on each plate to calculate absolute pg/mL values of cytokines for the samples assayed. Cases and controls were randomised across the plate. Plates were scanned on the Meso Scale Discovery MESO Quickplex SQ 120 reader at the Social, Genetic & Developmental Psychiatry Centre, Institute of Psychiatry, Psychology & Neuroscience, King’s College London. For the purpose of the current study, only data on IL-6, IL-10, and TNF-α were used as the DII has been previously validated against these inflammatory markers and they were available on the assay. For the results on group differences for the 36 inflammatory markers measured, see Keeler et al. [42].

### 2.3. Procedure

Eligible participants attended a single research session at the Institute of Psychiatry, Psychology & Neuroscience at King’s College London, lasting no longer than 1 and a half hours. After having their blood collected, participants had their height and body weight measured, from which BMI (kg/m^2^) was calculated, and body composition was assessed using a portable and non-invasive Inbody S10 machine, Biospace Co., Ltd. Participants then completed a questionnaire pack, including questions on demographic characteristics, clinical characteristics for AN and recAN participants, and the EPIC-Norfolk FFQ. Participants also completed questionnaires on mental health, data for which have been presented elsewhere [42] and will not be included in the present study.

### 2.4. Statistical Analysis

All statistical analyses were performed in SPSS [57]. For normally distributed data, means and standard deviations are presented, and for non-normally distributed data, median and interquartile ranges (25th and 75th percentile) are provided. One-way ANOVAs were used to assess group differences in demographic characteristics and nutrient values. For significant results, post-hoc analyses were performed to determine specific group differences. Post-hoc analyses were adjusted for multiple comparisons using the Bonferroni correction. Multivariable regression analyses were performed to assess the association between DII scores and inflammatory marker concentrations.

## 3. Results

### 3.1. Participants

A total of 133 participants were recruited. Data from 10 participants were excluded from the analyses for the following reasons: four HCs and one recAN participant were not within the required BMI range, two HCs reported regular recreational drug use, two HCs did not provide FFQ data, and one HC had tonsillitis in the previous week. Therefore, data on nutrient intake were available for a total of 123 participants. Table 1 summarises participant demographic and clinical characteristics.

### 3.2. Nutrient Intake

Table 2 presents nutrient intake of 25 micronutrients and macronutrients (as reported in the FFQ) with group comparisons. One-way ANOVAs and post-hoc pairwise comparisons revealed group differences for cholesterol, monounsaturated fats (MUFA), polyunsaturated fats (PUFA), total protein, and zinc (Table 2). Both current AN and recAN participants had significantly lower intake of cholesterol than HCs (*p* = 0.001 and *p* = 0.028 respectively); AN participants reported lower protein (*p* = 0.033) and zinc (*p* = 0.015) intake than HCs, and lower MUFA and PUFA intake than recAN (*p* = 0.030 and *p* = 0.050, respectively).

### 3.3. Dietary Inflammatory Index

While the DII score was lower in the recAN compared to the current AN and HC groups (AN = 0.56 [SD = 1.86], recAN = 0.07 [SD = 1.73], HC = 0.60 [SD = 2.08]), a one-way ANOVA and an ANCOVA controlling for calorie intake revealed no significant group differences in DII score (F (2, 121) = 0.553, *p* = 0.577 and F (2, 121) = 1.797, *p* = 0.170, respectively). The DII score for the 25 nutrients investigated in the whole sample ranged from −3.26 to +3.63.

### 3.4. Association between DII Score and Inflammatory Markers

Multivariable regressions controlling for age and calorie intake showed that DII score was significantly associated with concentrations of TNF-α in our AN sample (β = 0.404, t = 2.396, *p* = 0.021). However, DII score was not significantly related to any other cytokines in the AN group nor any cytokines in the recAN and HC groups.

## 4. Discussion

Individuals with AN report altered nutrient intake [7,8,9] and recent immunological studies in AN have found altered levels of cytokines compared to healthy individuals [39,58]. In this study, we aimed to examine group differences in nutrient intake and explore whether the inflammatory potential of an individual’s diet may be associated with inflammation.

### 4.1. Nutrient Intake

In our AN participants, we observed lower intake of cholesterol, protein, and zinc, compared to HCs, and MUFAs, compared to recAN participants. These findings could be explained by the food preferences of people with AN: research suggests that people with AN tend to prefer lower calorie options (e.g., avoid meat, dairy products, fried foods, and baked goods [59,60,61,62]) to prevent weight gain. It is important to note that we did not replicate well established findings, such as a reduced (total and saturated) fat intake in people with AN [8,61,63]. The lack of group differences may be accounted for by the large proportion of the AN sample who were receiving specialist ED treatment, as this aims to increase caloric consumption in a nutritionally balanced manner [61]. Methodological considerations associated with the FFQ may have also contributed to the findings, as will be discussed in Section 4.3.

We reported that both AN and recAN participants consumed significantly less cholesterol than HCs. This is unsurprising as people with AN tend to avoid foods that are high in cholesterol such as dairy products and meats [7,64]. Research on the lipid profile of AN patients has shown that they often exhibit hypercholesterolaemia [65,66]. This has been attributed to a diminished cholesterol and bile acid metabolism resulting from the reduced caloric intake [67,68] and suggests that, regardless of their dietary cholesterol intake, AN patients could be at risk of cardiovascular disease. With regards to recAN patients, research has found that they often make food choices based on their perceived health benefits [69]. Given the widely known health risks associated with high cholesterol, it is consistent with our recAN sample exhibiting lower cholesterol intake.

AN participants consumed significantly less protein than HCs in our study. Findings on consumption of protein in people with AN, compared to HCs, are mixed: some authors have reported increased protein intake [70,71], whereas others have reported lower intake [59,72,73]. Inadequate protein consumption can lead to decreased synthesis of visceral proteins, oedema, and muscle atrophy [74]. Indeed, oedema and muscle atrophy have been described in AN [75]. The lack of protein intake might have clinical implications for people with AN and a comorbid depressive or anxiety disorder. In AN, for example, recent studies found comorbidity rates of more than 50% for social anxiety disorder, about 40% for depression and 20–30% for generalized anxiety disorder [76,77]. Second-generation antidepressants such as selective serotonin reuptake inhibitors (SSRI) are the first-line pharmacological treatment for patients with depression and anxiety disorders [78,79]. However, SSRIs have not been found to have much benefit for depressive or anxious symptoms in the acute phase of AN [80]. The lack of proteins and amino acids has been suggested as a potential explanation because amino acids such as tryptophan are needed to produce neurotransmitters such as serotonin; and antidepressants, for example SSRIs, that act as reuptake inhibitors of neurotransmitters require the presence of neurotransmitters such as serotonin to be effective [81]. A comorbid depressive disorder may be a barrier to recovery from AN. Therefore, medications such as ketamine and esketamine which do not rely on the availability of amino acids have been suggested as treatment options for the treatment of a comorbid depressive disorder in malnourished patients with AN [82].

Zinc is perhaps the most studied micronutrient in AN, with previous research reporting deficient levels of zinc (<46 mcg/dL) in AN [59,72,83]. Our findings of lower zinc consumption in AN participants compared to HCs are consistent with some previous research [9]. However, some studies have reported no difference in zinc consumption, likely due to increased supplement use in AN [8]. Research has shown that people with AN are significantly more likely to be and/or have a history of being vegetarian, as compared to HCs [60,61]. As meat and fish are high in zinc, this may explain the present findings. The lack of zinc has also been suggested to play a role in the pathophysiology of depression and to contribute to therapy resistance during treatment with antidepressants; thus, zinc supplementation has been proposed as an adjunct to improve the efficacy of antidepressant treatment [84].

Compared to recAN participants, AN participants also consumed less MUFAs and PUFAs in this study. Indeed, people with current AN tend to restrict all types of dietary fats, whereas weight gain in AN has been associated with obtaining a higher percentage of total calories from fats, including unsaturated fats [85]. Overall, our results are in line with other studies showing essential fatty acid disturbances in current AN patients [86,87], as well as in chronically malnourished individuals [88,89]. However, it is perhaps surprising that group differences in total fat or saturated fat were not also identified, as it is well established that individuals with AN tend to prefer foods low in fat [8,64].

### 4.2. Dietary Inflammatory Index

Our analyses did not reveal any significant differences in DII score between the current AN, recAN and HC groups, despite the recAN participants having a lower DII score than the other groups. Given the few differences we identified in nutritional intake between groups, and the narrow range of inflammatory scores in the whole sample, this is perhaps to be expected.

We also explored whether DII score may be associated with cytokine concentrations in each of our groups. Inflammatory marker analysis revealed that DII score was associated with concentrations of TNF-α but only in the AN group (when controlling for age and calorie intake), such that a higher DII score was associated with increased concentrations of TNF-α. However, no other associations between DII score and inflammatory marker concentrations were found. Therefore, it may suggest that factors other than dietary intake regulate cytokines in current and recovered AN and be responsible for the alterations reported previously [5,6]. Indeed, there is likely a combination of factors that contribute to the observed altered concentrations of inflammatory markers in currently unwell AN patients. Alternative factors could include stress, genetics, and comorbid psychiatric disorders, as well as specifically AN-related factors like current recovery and refeeding status, recent weight gain, and current ED behaviours (e.g., self-starvation and compensatory mechanisms). Alongside this, other behavioural factors could also contribute to heightened concentrations of inflammatory markers observed in AN patients. Often correlated, health behaviours such as physical activity, tobacco smoking, and sleeping patterns [90,91] can impact on inflammatory status [92], and have previously been shown to be altered in people with eating disorders [93,94,95].

### 4.3. Strengths and Limitations

This is the first study to assess inflammatory potential of dietary intake in people with AN. However, the sample was relatively small, particularly in the recAN group, within which we performed multiple comparisons. Our sample size did not allow us to subdivide our AN participants according to their AN subtype (binge-eating/purging type or restricting type), which would have been of interest given likely differences in dietary patterns between the AN subtypes. Additionally, our sample was heterogeneous in demographic and clinical characteristics, including disease duration which ranged from 3 months to 35 years. Further issues and uncertainties to consider are that eating more food tends to be associated with lower DII scores, that findings seem to vary between studies depending on patterns of food intake within individual populations, and that there is often limited eating pattern variability within control groups [56]. Therefore, our results need to be interpreted with caution.

There are further methodological considerations that may have contributed to our findings, namely, the inherent strengths and limitations associated with the use of FFQs. The FFQ represents a good option for capturing dietary information as it is simple to self-administer, relatively low-cost, and may be a better representation of usual dietary patterns than 24-h recall or a few days of observation. However, there are also several limitations to this method of collecting data on nutritional intake. The EPIC-Norfolk FFQ requires participants to recall the frequency and portion size consumption over the last year. This is cognitively demanding and is often biased by their present dietary intake and patterns. Additionally, for AN participants who have been in treatment, it may have been difficult to record an average intake when their diet may have altered during this time frame, as nutrition restoration is a key component of treatment for AN. Further, food portion estimation is frequently imprecisely estimated and quantified: research has shown that people with AN tend to overestimate energy intake perhaps due to over-reporting of caloric intake; in contrast, HCs tend to consistently under-report caloric intake [72,96,97]. Hence, dependence on participant recall makes FFQs amenable to misrepresenting true dietary intake [98]. Finally, the FFQ is limited to a specific list of food items, which could be considered outdated given that it was designed approximately 20 years ago. Dietary habits have changed considerably over the last two decades [99,100]. For example, the list does not include non-dairy milk alternatives, consumption of which was reported by a large proportion of our contemporary participants.

## 5. Conclusions

In participants with current AN, we identified significantly lower intake of cholesterol, protein, and zinc, compared to HCs and MUFAs and PUFAs compared to participants recovered from AN. The DII score did not significantly differ between groups. Therefore, the findings from this study suggest that it is unlikely that a pro-inflammatory diet accounts for the alterations in cytokines and other inflammatory markers that have been observed in people with current AN. As this is the first study to assess dietary inflammation in AN, future research should further explore the use of the DII in samples with current and recovered AN, using multiple 24-h dietary recalls (to avoid problems associated with averaging intake over a long period of time).

## Figures and Tables

**Table 1:** Participant demographic and clinical characteristics with group comparisons.

	HC *n* = 49	AN *n* = 51	RecAN *n* = 23	Group Comparisons
Demographic characteristics				
Age [years] [median (IQR ^a^)]	22.5 (20.3, 25.8)	24 (21.0, 30.0)	24 (21.0, 30.0)	*H* (2) = 4.003 *p* = 0.135
Ethnicity [Caucasian/BAME] [*n*]	25/24	45/6	22/1	F (2) = 5.96 *p* = 0.003
Current smoker [*n*]	7	8	3	F (2) = 0.068 *p* = 0.93
BMI [kg/m^2^] [median (IQR ^a^)]	21.0 (19.6, 22.5)	16.1 (15.1, 17.0)	20.7 (19.6, 21.3)	*H* (2) = 84.121 *p* < 0.001
Body fat [%] [mean ± SD]	23.9 ± 5.2	12.0 ± 5.2	22.3 ± 6.3	F (2) = 69.33 *p* < 0.001
Clinical characteristics				
AN subtype [AN-R/AN-BP] [*n*]		45/6		
Current treatment [none/outpatient/inpatient] [*n*]		20/30/1		
Disease Duration [years]		5.57		
Current antidepressant use [*n*]	0	21	6	F (2) = 13.88 *p* < 0.001
Current antipsychotic use [*n*]	0	6	2	F (2) = 3.21 *p* = 0.04

Statistically significant group comparisons at *p* < 0.05 are highlighted in bold. ^a^ 25th and 75th percentile reported. Abbreviations: HC = healthy controls; AN = anorexia nervosa; recAN = recovered anorexia nervosa; IQR = interquartile range; BAME = Black, Asian, and minority ethnic; *n* = number of observations; BMI = body mass index; AN-R = anorexia nervosa restricting type; AN-BP = anorexia nervosa binge-eating/purging type.

**Table 2:** Between groups comparisons of individual nutrient and food component intake.

Nutrient	AN [SD] (*n* = 51)	RecAN [SD] (*n* = 23)	HC [SD] (*n* = 49)	Group Comparison	Post-Hoc Pairwise Comparison
Alcohol [g]	2.22 [4.35]	4.85 [7.05]	3.13 [3.37]	F (2) = 2.561 *p* = 0.081	HC—AN: *p* = 0.978 HC—RecAN: *p* = 0.436 AN—RecAN: *p* = 0.077
β-carotene [μg]	5239.39 [4561.55]	5701.02 [4846.22]	4387.41 [3068.63]	F (2) = 0.973 *p* = 0.381	HC—AN: *p* = 0.900 HC—RecAN: *p* = 0.619 AN—RecAN: *p* = 1.000
Total carbohydrate [g]	204.37 [108.67]	232.42 [99.08]	218.22 [118.02]	F (2) = 0.539 *p* = 0.585	HC—AN: *p* = 1.000 HC—RecAN: *p* = 1.000 AN—RecAN: *p* = 0.947
Cholesterol [mg]	168.56 [132.23]	181.90 [139.96]	294.07 [208.61]	F (2) = 7.72 *p* = 0.001	HC—AN: *p* = 0.001 HC—RecAN: 0.028 AN—RecAN: *p* = 1.000
Energy [kcal]	1553.64 [739.79]	1928.31 [875.55]	1823.68 [950.70]	F (2) = 2.0 *p* = 0.140	HC—AN: *p* = 0.350 HC—RecAN: *p* = 1.000 AN—RecAN: *p* = 0.250
Total fat [g]	57.43 [35.27]	81.07 [44.25]	72.16 [41.05]	F (2) = 3.375 *p* = 0.038	HC—AN: *p* = 0.192 HC—RecAN: p = 1.000 AN—RecAN: *p* = 0.055
Folate [μg]	323.52 [183.11]	375.31 [209.03]	310.05 [151.86]	F (2) = 1.094 *p* = 0.338	HC—AN: *p* = 1.000 HC—RecAN: *p* = 0.440 AN—RecAN: *p* = 0.736
Englyst fibre [g]	20.40 [11.41]	23.28 [13.94]	18.80 [10.47]	F (2) = 1.176 *p* = 0.312	HC—AN: *p* = 1.000 HC—RecAN: *p* = 0.385 AN—RecAN: *p* = 0.973
Iron [mg]	10.86 [5.21]	13.22 [6.54]	11.93 [5.90]	F (2) = 1.380 *p* = 0.256	HC—AN: *p* = 1.000 HC—RecAN: *p* = 1.000 AN—RecAN: *p* = 0.316
Magnesium [mg]	319.62 [129.58]	388.72 [202.81]	337.13 [170.24]	F (2) = 1.457 *p* = 0.237	HC—AN: *p* = 1.000 HC—RecAN: *p* = 0.627 AN—RecAN: *p* = 0.274
Monounsaturated fat [g]	21.03 [12.46]	30.93 [18.35]	27.75 [15.87]	F (2) = 4.279 *p* = 0.016	HC—AN: *p* = 0.083 HC—RecAN: *p* = 1.000 AN—RecAN: *p* = 0.030
Niacin (vitamin B_3_) [mg]	20.51 [10.60]	22.32 [11.79]	21.97 [11.63]	F (2) = 0.299 *p* = 0.742	HC—AN: *p* = 1.000 HC—RecAN: *p* = 1.000 AN—RecAN: *p* = 1.000
Total protein [g]	63.91 [31.43]	73.61 [34.67]	83.85 [46.41]	F (2) = 3.325 *p* = 0.039	HC—AN: *p* = 0.033 HC—RecAN: *p* = 0.890 AN—RecAN: *p* = 0.959
Polyunsaturated fat [g]	11.91 [7.34]	17.01 [10.88]	13.22 [8.01]	F (2) = 2.965 *p* = 0.055	HC—AN: *p* = 1.000 HC—RecAN: *p* = 0.227 AN—RecAN: *p* = 0.050
Riboflavin (vitamin B_2_) [mg]	1.87 [1.12]	1.97 [1.06]	1.82 [0.91]	F (2) = 0.174 *p* = 0.841	HC—AN: *p* = 1.000 HC—RecAN: *p* = 1.000 AN—RecAN: *p* = 1.000
Saturated fatty acids [g]	19.54 [14.91]	26.64 [16.07]	24.87 [15.50]	F (2) = 2.302 *p* = 0.104	HC—AN: *p* = 0.256 HC—RecAN: *p* = 1.000 AN—RecAN: *p* = 0.205
Selenium [μg]	50.83 [30.96]	54.35 [27.31]	63.16 [34.12]	F (2) = 1.954 *p* = 0.146	HC—AN: *p* = 0.162 HC—RecAN: *p* = 0.819 AN—RecAN: *p* = 1.000
Thiamin (Vitamin B_1_) [mg]	1.51 [0.81]	1.94 [1.11]	1.59 [0.82]	F (2) = 1.957 *p* = 0.146	HC—AN: *p* = 1.000 HC—RecAN: *p* = 0.336 AN—RecAN: *p* = 0.163
Retinol (Vitamin A) [μg]	251.89 [368.00]	313.50 [358.19]	421.35 [398.41]	F (2) = 2.532 *p* = 0.084	HC—AN: *p* = 0.081 HC—RecAN: *p* = 0.786 AN—RecAN: *p* =1.000
Vitamin B_6_ [mg]	1.83 [0.89]	2.12 [1.01]	2.23 [1.07]	F (2) = 2.168 *p* = 0.119	HC—AN: *p* = 1.000 HC—RecAN: *p* = 0.130 AN—RecAN: *p* = 0.735
Vitamin B_12_ [μg]	4.27 [3.69]	3.51 [2.87]	5.66 3.61]	F (2) = 3.527 *p* = 0.032	HC—AN: *p* = 0.151 HC—RecAN: *p* = 0.051 AN—RecAN: *p* = 1.000
Vitamin C [mg]	146.15 [90.44]	142.01 [91.63]	123.88 [69.78]	F (2) = 0.961 *p* = 0.385	HC—AN: *p* = 0.548 HC—RecAN: *p* = 1.000 AN—RecAN: *p* = 1.000
Vitamin D [μg]	2.04 [2.32]	2.11 [1.82]	2.73 1.98]	F (2) = 1.508 *p* = 0.225	HC—AN: *p* = 0.309 HC—RecAN: *p* = 0.720 AN—RecAN: *p* = 1.000
Vitamin E [mg]	12.58 [5.53]	15.44 [7.89]	12.45 [7.06]	F (2) = 1.813 *p* = 0.168	HC—AN: *p* = 1.000 HC—RecAN: *p* = 0.233 AN—RecAN: *p* = 0.268
Zinc	7.27 [3.23]	8.67 [4.35]	9.76 [5.26]	F (2) = 4.115 *p* = 0.019	HC—AN: *p* = 0.015 HC—RecAN: *p* = 0.971 AN—RecAN: *p* = 0.606

Abbreviations: AN = anorexia nervosa, HC = healthy controls, RecAN = recovered anorexia nervosa, *n* = number of observations.

## Data Availability

The data presented in this study are available on request from the corresponding author. The data are not publicly available as they are still being used for analysis and manuscript writing.
